# Effects of a new respiratory muscle training device in community-dwelling elderly men: an open-label, randomized, non-inferiority trial

**DOI:** 10.1186/s12877-022-02828-8

**Published:** 2022-02-24

**Authors:** Sang Hun Kim, Myung-Jun Shin, Jang Mi Lee, Sungchul Huh, Yong Beom Shin

**Affiliations:** 1 Department of Rehabilitation Medicine, Biomedical Research Institute, Pusan National University Hospital, Busan, Republic of Korea; 2 Department of Rehabilitation Medicine, Biomedical Research Institute, Pusan National University Hospital and Pusan National University School of Medicine, Busan, Republic of Korea; 3 Busan Center for infectious Disease Control and Prevention, Pusan National University Hospital, Busan, Republic of Korea; 4 Department of Rehabilitation Medicine, Research Institute for Convergence of Biomedical Science and Technology, Pusan National University Yangsan Hospital, Yangsan, Republic of Korea

**Keywords:** Breathing exercises, Diaphragm, Exercise therapy, Maximal respiratory pressure, Respiratory function test, Respiratory muscles

## Abstract

**Background:**

Respiratory muscle training (RMT) has various clinical benefits in older adults; however, the low adherence to training remains a challenging issue. The present study aimed to confirm the efficacy of a new device that combines inspiratory muscle training and a positive expiratory pressure (IMT/PEP) compared to that of a Threshold IMT device (Philips Respironics Inc), and to determine whether home-based training differed from rehabilitation center training.

**Methods:**

This four-arm, multicenter, parallel, non-inferiority trial randomized 80 active community-dwelling older men (mean age = 72.93 ± 5.02 years) to center-based groups (new IMT/PEP device or Threshold IMT device; 16 supervised sessions) or home-based groups (new IMT/PEP device or Threshold IMT device; 2 supervised sessions and individual sessions). Participants in all groups performed RMT twice a day for 8 weeks. Assessments were performed at baseline and post-training. The primary outcomes were maximum inspiratory pressure and maximal expiratory pressure. The secondary outcomes included forced vital capacity and forced expiratory volume in the first second, peak cough flow, diaphragm thickness, VO_2_ peak, the International Physical Activity Questionnaire score, electromyographic activities of the sternocleidomastoid muscle, and skeletal muscle mass and phase angle as measured by bioimpedance analysis. In addition, rates of adherence to each protocol were also compared.

**Results:**

Among all groups, the maximal inspiratory pressure was improved post-training, while the maximal expiratory pressure showed improvement only in the IMT/PEP groups. The overall non-inferiority of the IMT/PEP device was thus validated. A statistically significant improvement in diaphragm thickness was found. However, no consistent improvement was shown in other secondary outcomes. No significant difference in training adherence rate between protocols was observed (mean adherence rate of 91–99%).

**Conclusion:**

Compared to the Threshold IMT, the new IMT/PEP device did not result in a significant difference in maximal inspiratory pressure but did improve maximal expiratory pressure in older men. The IMT/PEP device’s improved usability, which is associated with exercise adherence, provided distinct advantages in this cohort. If proper education is first provided, home-based RMT alone may provide sufficient effects in older individuals.

**Trial registration:**

This trial was registered in the database cris.nih.go.kr (registration number KCT0003901) on 10/05/2019.

## Background

Physiological pulmonary function changes in older individuals are characterized by reduced lung elasticity, respiratory muscle strength, and chest wall compliance [1, 2]. Sarcopenia is defined as the age-related loss of skeletal muscle mass, muscle strength, and reduced physical performance [3]. In addition, respiratory muscle strength decreases in older individuals with sarcopenia [4]. These physiological changes in older individuals make it difficult to maintain physical activity, which is essential for maintaining a healthy lifestyle [5].

**Fig. 1:**
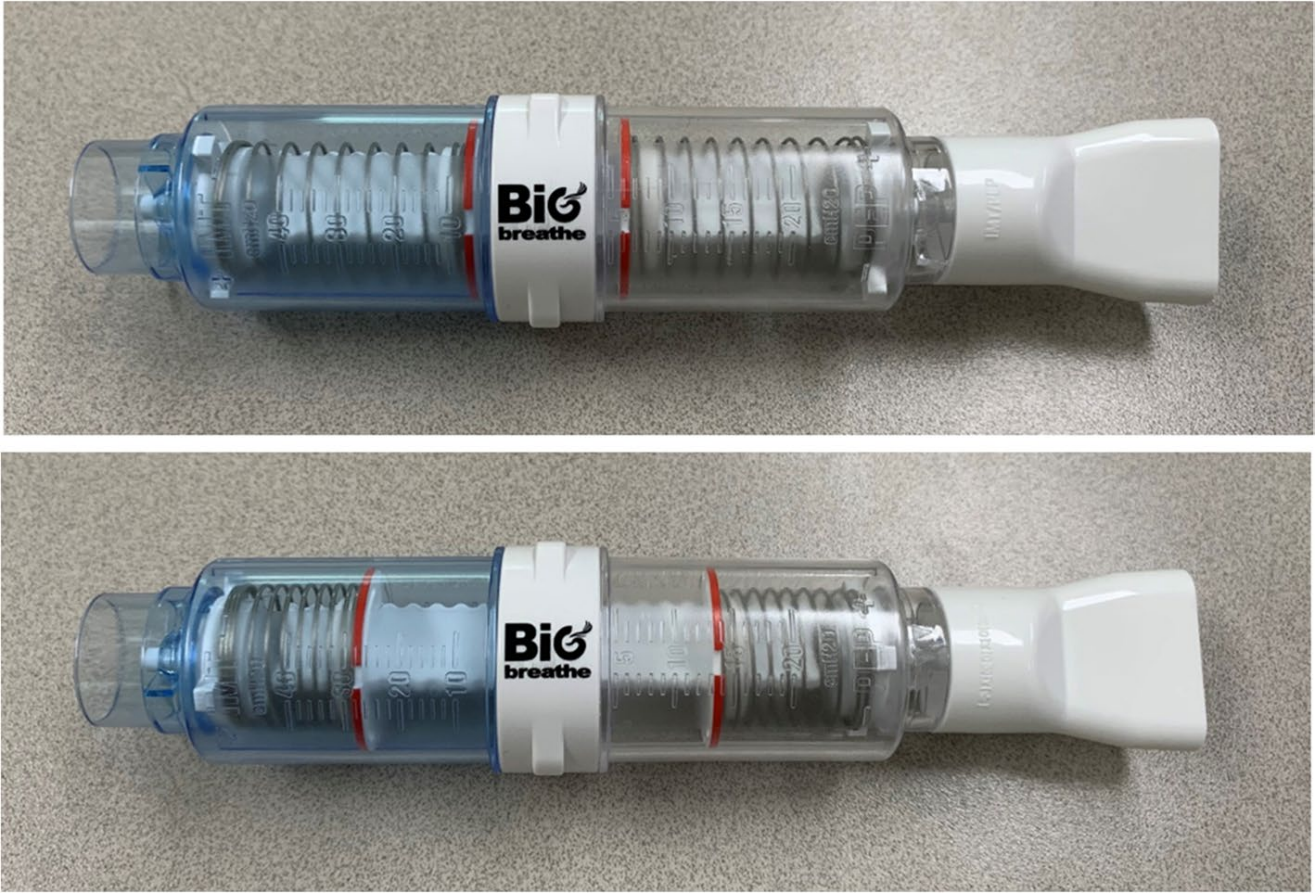
The new combined IMT/PEP device (numbers presented are in mm)

The weakness of respiratory muscles in older individuals can increase the prevalence of diseases and disability [6]. Therefore, various exercises have been proposed to improve physical performance in older individuals [7, 8]. Several clinical effects of respiratory muscle training (RMT), such as a strengthened diaphragm and improved aerobic capacity and coughing ability, have been reported in older individuals [9, 10]. RMT has improved physical performance in less-fit individuals, which includes older individuals [11]. However, low adherence to pulmonary rehabilitation exercise, including RMT, remains problematic [12]. For these reasons, we have designed a new device that combines inspiratory muscle training (IMT) and positive-expiratory pressure (PEP) for enhancing exercise adherence and usability. The new device enables a two-way simultaneous threshold RMT (IMT/PEP, GH INNOTEK, Busan, South Korea; Fig. 1), which has already proven its superior effect among various RMTs [11]. As the era of the COVID-19 pandemic continues, poor access to training centers and decreased completion of exercises are challenges in older individuals as well as in patients with respiratory diseases [13, 14]. Alternatively, home-based exercises could be effective to achieve adequate adherence, in the same way as conventional center-based exercises [15, 16]. However, the effect and adherence to center-based RMT and home-based RMT have not yet been compared.

The aim of this present study was to confirm the efficacy of the new IMT/PEP device and to determine whether home-based training was better than rehabilitation center-based training in terms of improving the training adherence rate and effect. Therefore, in this randomized clinical trial, we investigate whether RMT using the new combined IMT/PEP device was non-inferior to that using the existing Threshold IMT (Philips Respironics Inc., Murrysville, PA, USA) device, and whether a home-based RMT program had non-inferior effects compared to center-based RMT.

## Methods

### Study design

This study was a four-group, multicenter, randomized, parallel, non-inferiority trial with concealed allocation and assessor blinding. All participants provided written informed consent. Ethics approval was obtained from the Institutional Review Boards (IRBs) of Pusan National University Hospital (IRB No. 1903–028-076) and Pusan National University Yangsan Hospital (IRB No. 03–2019-006). All procedures of the study were performed in accordance with the amended Declaration of Helsinki. This study was registered at Clinical Research Information Service (No. KCT0003901**)**.

### Recruitment and sample size

Participants were recruited through a research recruitment flyer from welfare centers for older individuals in Busan, South Korea, from April 2019 to August 2020. Community-dwelling men over 60 years of age who were able to walk without a mobility aid were included. Exclusion criteria were as follows: known cardiopulmonary diseases that cause chest pain or dyspnea during activity; uncontrolled musculoskeletal pain; participation in other clinical trials within the last 4 weeks; and the presence of diseases, such as glaucoma, aneurysm, or pulmonary artery hypertension, which would prohibit the Valsalva maneuver [17]. The level of dyspnea during activity by which individuals were excluded from participation was more than 2 points on the modified Medical Research Council dyspnea scale [18]. The sample size was determined by calculations performed on data collected from a previous study [9]. Thus, 17 participants per group were necessary, based on a mean difference in the maximum inspiratory pressure (MIP) between exercises, with an alpha-risk of 0.05 and a beta-risk of 0.20 in a two-tailed test. To allow a 15% dropout rate, a sample size of 20 participants per group was finally determined.

### Randomization and interventions

Prior to randomization, all participants underwent screening and familiarization with the training protocol before the baseline outcomes were evaluated. A block-randomization process was performed with a block-size of 16, using computer-generated random allocation in Excel 2016 (Microsoft, Redmond, WA, USA). Random allocation was generated by an individual who did not participate in the study. The participants were randomly assigned in a 1:1:1:1 manner into the following groups, depending on the device allocated and the training site: IMT/PEP in the rehabilitation center (Group N-C), Threshold IMT in the rehabilitation center (Group I-C), IMT/PEP at home (Group N-H), and Threshold IMT at home (Group I-H).

For all participants, several practice tests were performed to correct possible training and learning effects, before the tests were conducted. All evaluations and training were conducted by different blinded researchers. The new IMT/PEP device used in this study had a threshold IMT range of 10–40 cmH_2_O and PEP of 5–20 cmH_2_O, with a resolution of 2 cmH_2_O. A variable loading can be set on the IMT/PEP device, providing flow-independent resistance to inspiration or expiration, by using two spring-loaded one-way valves. The valves of this device only opened when the pressure generated by the participant exceeded the set spring tension during inspiration and expiration. This concept is similar to that of the existing individual Threshold IMT and Threshold PEP devices (Philips Respironics Inc., Murrysville, PA, USA), but is designed in a way that allows simultaneous training of both IMT and PEP in one breathing cycle. Groups N-C and I-C visited the rehabilitation center twice a week to undergo supervised training over a period of 8 weeks. They also performed self-training at an individualized intensity twice a day at home for 8 weeks. Groups N-H and I-H performed the same RMT twice a day at home for 8 weeks, with only two supervised training sessions. For all participants, one self-training session was omitted (on the day of supervised training). A telephone interview was conducted 4 weeks after enrollment to confirm whether there were any problems with the training or device for the participants in Groups N-H and I-H.

The inspiratory threshold for each device was set to 40% of the initial MIP of each participant. The maximum threshold of the device was 40 cmH_2_O. Considering that the average maximal expiratory pressure (MEP) was above 100 cmH_2_O for those over 60 years of age [19], the expiratory threshold was set to 20 cmH_2_O (the maximum load of the device) for groups using the combined IMT/PEP device (Groups N-C and N-H). All participants were instructed to inspire, from the residual volume, at a constant intensity and strength, to the point where they exceeded the threshold pressure. When they reached vital capacity, they held their breath for several seconds and then exhaled at a constant intensity for as long as possible. Each RMT set consisted of 10 deep and forceful breaths against the threshold pressure of the device. Participants were required to perform 10 sets twice a day, with 2 min of rest after each set. During the first training session, two RMT sets were practiced at half the target threshold to allow the patient to adapt to the devices. All participants kept a home exercise diary, which was used to check their training adherence, and all training in the rehabilitation center was conducted under the supervision of an experienced physiotherapist.

### Measurements

All participants were assessed on the date of their first visit and at 1 week after completing the training program by an assessor blinded to the group allocation. Clinical and demographic data were collected, and the level of physical activity was evaluated using the International Physical Activity Questionnaire (IPAQ), adapted to older individuals [20]. The primary outcomes in this study were MIP and MEP, and the secondary outcomes were forced vital capacity (FVC) and forced expiratory volume in the first second (FEV_1_), peak cough flow (PCF), ultrasound-based diaphragm thickness, predicted VO_2_ peak, IPAQ score, electromyography activities of the sternocleidomastoid muscle, and skeletal muscle mass and phase angle by bioimpedance analysis (BIA).

### Primary outcomes

MIP, MEP, and pulmonary functions were evaluated in a standardized method using a desktop spirometer Pony FX (Cosmed, Rome, Italy) [21, 22]. MIP and MEP, reflect inspiratory and expiratory muscle strength, respectively. Measurements were obtained, with participants in a sitting position, using a flange-type mouthpiece. The MIP was acquired from one maximal inspiration, starting from close to the residual volume. The MEP was obtained from the maximal expiration, starting from close to the vital capacity. At least five measurements were obtained, and when reproducible measurements with a difference of less than 10% were obtained, the highest three measurements were recorded [23]. Prediction of MIP and MEP was calculated using the following reference eqs [19].:

$${\displaystyle \begin{array}{c}\mathrm{Male}\ \mathrm{MIP}\ \mathrm{reference}=120-\left(0.41\times \mathrm{age}\right)\\ {}\mathrm{Male}\ \mathrm{MEP}\ \mathrm{reference}=174-\left(0.83\times \mathrm{age}\right)\end{array}}$$

### Secondary outcomes

The PCF was measured with a Micro Peak-flow meter (Micro Medical, Calabasas, CA, USA.) The result recorded was the maximum value obtained from three trials of a short and forceful expiration after a maximum inspiration [24]. FVC and FEV_1_ were measured from maximum inspiration and expiration after taking three normal breaths, in accordance with the following reference eqs [25]..

$${\displaystyle \begin{array}{c}\mathrm{FVC}\ \mathrm{in}\ \mathrm{male}\ \left(\mathrm{liter}\right)=-4.8434-0.00008633\times {\mathrm{Age}}^2\kern0.5em +\kern0.5em 0.05292\times \mathrm{Height}\ \left(\mathrm{cm}\right)+\kern0.75em 0.01095\times \mathrm{Weight}\ \left(\mathrm{kg}\right)\\ {}\mathrm{FEV}1\ \mathrm{in}\ \mathrm{male}\ \left(\mathrm{liter}\right)=-3.4132-0.0002484\times {\mathrm{Age}}^2+\kern0.5em 0.04578\times \mathrm{Height}\ \left(\mathrm{cm}\right)\end{array}}$$

Ultrasound (Z.ONE, ZONARE, Mountain View, CA, USA) was used to measure diaphragm thickness, between the 8th and 9th ribs of the anterior and mid-axillary lines, using a 12-MHz linear probe in B-mode [26]. Participants were instructed to breathe quietly and spontaneously in the supine position, and the right diaphragm thickness at the end of quiet expiration (Texp) and at the end of quiet inspiration (Tins) was measured in mm. A total of five measurements were obtained, and the three values, excluding the maximum and minimum values, were recorded.

The Chester step test is an effective and simple method for evaluation of aerobic capacity [27]. As a submaximal test, steps can be performed at various heights with both feet according to a metronome rhythm. In this study, a 20-cm step box was used for participants over 60-years-old. In stage 1, steps were performed at a speed of 60 beats per minute, and the speed was increased by 20 beats per minute every 2 min. Heart rate, oxygen saturation, and the Borg Category/Ratio-10 Dyspnea Scale® were measured before and at the end of each stage. The predicted oxygen consumption based on the heart rate at each stage was calculated [28]. Phase angles and skeletal muscle mass were quantified in participants using a segmental multi-frequency BIA system (S10, InBody Co., Ltd., Seoul, South Korea). Touch-type electrodes were attached between the participants’ ankles and on the middle finger and thumb of both hands. Participants rested in a supine position for several minutes before the BIA measurements were taken. The phase angles of each segment of the body were automatically calculated at frequencies of 5, 50, and 250 kHz by the BIA system’s software. Among the many variables, we analyzed the 50-kHz whole-body phase angle.

To measure the muscle activity required for the target pressures, during the first and last supervised training for effective RMT, a surface electromyography (sEMG) device was also applied. The single-channel sEMG device (PSL-EMG-Tr1; PhysioLab Co., Ltd., Busan, South Korea) was set to a sampling rate of 30,000 Hz, and signals were amplified within a 3–2000-Hz bandwidth [29]. Conductive adhesive hydrogel electrodes (Covidien, Minneapolis, MN, USA) were placed parallel to the left sternocleidomastoid (SCM) fibers according to the recommendation [30]. Before the sEMG measurement, the participants sat upright on an adjustable-height stool, with a neutral head posture, maintaining the normal curvature of the spine. Afterward, they held the RMT training device with the right hand, and muscle activity was measured during RMT. The muscle activity of the SCM used in forceful inspiration was presented in real-time through a tablet screen, and feedback on the proper use of respiratory muscle during training was available. The root mean square (RMS) reflected the activities of the motor unit in muscle contraction [31]. The RMS values of the recorded sEMG obtained from the initial feedback training and follow-up test were used for analysis. To standardize the measurement, the mean RMS of the left SCM muscle obtained in the fifth set of training was calculated for all participants. The mean RMS obtained in the middle 2s of each inspiration was analyzed.

### Statistical analysis

A minimal clinically important significance (MCID) of 11 cmH_2_O in the MIP of the Threshold IMT group was used to confirm non-inferiority [31]. The IMT/PEP groups were considered non-inferior to the Threshold IMT groups if the upper MIP limit did not exceed the 95% confidence interval in the Threshold IMT group. In addition, variables were compared between the center-training and the home-training groups to identify differences according to the training protocol when using the same device. The result of the baseline characteristics and analyzed outcomes are presented as mean ± standard deviation. We used an intention-to-treat approach for the primary analysis. One-way analysis of variance was used to identify demographic differences among the four groups. Normality of data distribution was verified through the Shapiro–Wilk test. Comparison of values pre- and post-training in each group were performed via the paired *t*-test and the Wilcoxon signed-rank test. The independent t-test and Mann–Whitney test were used to compare values between groups. A *p*-value < 0.05 was considered statistically significant. All statistical analyses were performed using SPSS Statistics for Windows (version 22, Chicago, IL, USA).

## Results

**Fig. 2:**
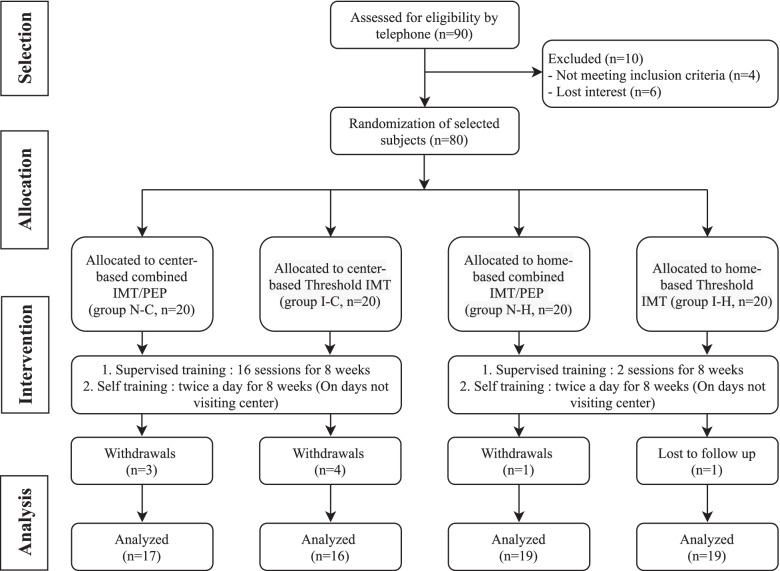
Flow diagram of the study participants

Eighty participants were randomized equally into four groups. Groups N-C and N-H used the IMT/PEP device and Groups I-C and I-H used the Threshold IMT (Fig. 2). During the 8-week intervention, seven (17.5%) participants in the center-based groups and two (5%) in the home-based groups withdrew from the study. Reasons for the dropout included a loss to follow-up, loss of interest in training, or participants being too busy. Table 1 presents the demographics and baseline outcomes of each group. There were no statistically significant differences in demographic variables among the four groups.

**Table 1:** Demographics and baseline characteristics of the participants

Parameters	Group N-C (n = 20)	Group I-C (*n* = 20)	Group N-H (*n* = 20)	Group I-H (*n* = 20)	*P* value
Age (years)	73.00 ± 5.36	73.86 ± 3.46	74.32 ± 4.82	71.95 ± 5.09	0.374
Weight (kg)	68.20 ± 8.94	67.55 ± 9.66	67.70 ± 10.44	71.91 ± 9.24	0.574
Height (m)	1.64 ± 0.04	1.63 ± 0.06	1.66 ± 0.06	1.67 ± 0.04	0.434
BMI (kg/m^2^)	25.18 ± 3.08	24.94 ± 2.66	24.57 ± 2.75	25.78 ± 2.98	0.684
FVC (L)	3.22 ± 0.51	3.19 ± 0.57	3.04 ± 0.46	3.25 ± 0.58	0.403
FVC (% predicted)	99.23 ± 14.28	100.28 ± 16.20	94.15 ± 16.13	95.36 ± 13.89	0.583
FEV_1_ (L)	2.48 ± 0.41	2.42 ± 0.59	2.31 ± 0.49	2.46 ± 0.59	0.653
FEV_1_ (% predicted)	112.17 ± 16.71	110.35 ± 27.18	106.31 ± 24.73	105.84 ± 21.09	0.788
FEV_1_/FVC (%)	76.17 ± 5.55	74.57 ± 9.61	75.57 ± 11.28	74.89 ± 9.71	0.734
PCF (L/min)	438.82 ± 74.57	414.28 ± 96.13	426.31 ± 87.63	424.21 ± 111.22	0.962
MIP (cmH_2_O)	93.05 ± 23.81	78.35 ± 15.43	77.99 ± 18.01	85.33 ± 30.11	0.316
MIP (% predicted)	103.38 ± 26.78	87.42 ± 17.68	87.11 ± 20.23	94.13 ± 32.45	0.313
MEP (cmH_2_O)	82.54 ± 24.11	96.07 ± 21.53	92.07 ± 18.03	99.00 ± 29.27	0.100
MEP (% predicted)	72.70 ± 20.61	85.26 ± 18.98	81.91 ± 15.42	86.55 ± 24.90	0.119
VO_2_ peak (ml/kg/min)	33.85 ± 5.55	29.01 ± 5.58	35.76 ± 9.45	34.52 ± 6.29	0.466
Adherence rate (% sessions completed)	91.94 ± 21.93	99.50 ± 1.87	92.37 ± 14.35	92.05 ± 20.30	0.491
RMS (uV)	49.69 ± 20.56	46.27 ± 19.86	55.79 ± 31.96	42.37 ± 22.27	0.416
Right diaphragm thickness at end inspiration (mm)	2.90 ± 0.82	3.24 ± 0.94	2.63 ± 0.77	2.88 ± 0.59	0.207
SMI (kg/m^2^)	8.88 ± 0.89	8.90 ± 0.73	8.74 ± 0.72	9.13 ± 0.69	0.453
Bioimpedance-derived phase angle	5.84 ± 0.71	5.84 ± 0.55	5.90 ± 0.50	6.16 ± 0.51	0.178
IPAQ (MET-min/week)	2998.23 ± 3135.65	2365.92 ± 2286.87	3071.89 ± 2164.56	3672.10 ± 4046.08	0.455
IPAQ (activity level)	2.11 ± 0.48	2.21 ± 0.57	2.36 ± 0.49	2.10 ± 0.73	0.422

Continuous variables are reported as mean ± standard deviation

***Abbreviations*:** *BMI* body mass index, *FVC* forced vital capacity, *FEV*_*1*_ forced expiratory volume in the first second, *PCF* peak cough flow, *MIP* maximal expiratory pressure, *MEP* maximal expiratory pressure, *VO*_*2*_ *peak* peak oxygen uptake, *RMS* root mean square, *SMI* skeletal muscle index, *IPAQ* International Physical Activity Questionnaire

### MIP and MEP

**Fig. 3:**
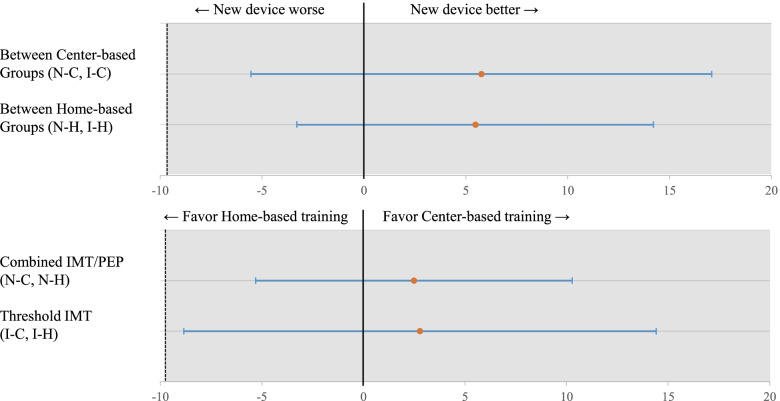
Non-inferiority plot of MIP. Difference between devices (above) and protocols (below) in the change of MIP from weeks 0 to 8. Error bars indicate the 95% confidence intervals, and the shaded area indicates the non-inferiority zone

**Fig. 4:**
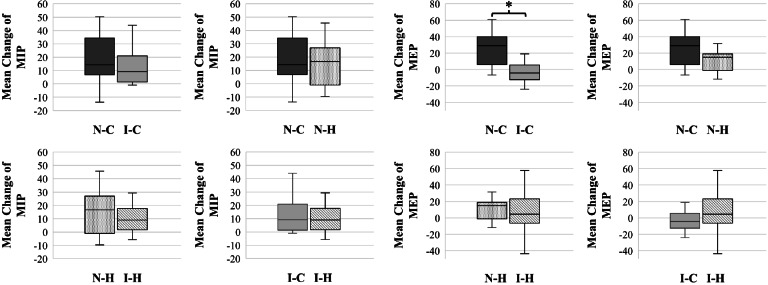
Mean changes in MIP and MEP after 8 weeks of training between devices or protocols. *P = 0.002

In the pre- and post-assessment of the 8-week intervention, the MIP and MEP were significantly improved in Groups N-C (*P* = 0.001 and *P* < 0.001 respectively) and N-H (*P* < 0.001 and *P* = 0.008, respectively), but in Groups I-C (P = 0.001 and *P* = 0.874) and I-H (*P* < 0.001 and *P* = 0.136), significant improvements were identified only in the MIP (Table 2). As illustrated in Fig. 3, the lower limit of the 95% confidence interval was within the non-inferiority margin of the MIP between Groups N-C/I-C and N-H/I-H. Specifically for the MIP, improvements by training were identified in all groups, and there were no statistically significant differences between devices and training protocols. In the MEP, statistically significant differences were identified only between Groups N-C and I-C (*P* = 0.002) (Table 2, Fig. 4).

**Table 2:** Mean changes and mean differences of MIP and MEP after training within and between groups

	Group N-C (*n* = 17)		Group I-C (*n* = 16)		Group N-H (*n* = 19)		Group I-H (*n* = 19)	
Outcomes	Change from baseline	*P* value	Change from baseline	*P* value	Change from baseline	*P* value	Change from baseline	*P* value
		ES		ES		ES		ES
MIP (cmH_2_O)	18.03 ± 17.87 (9.15 to 26.9)	0.001*	12.66 ± 12.76 (5.86 to 19.46)	0.001*	15.65 ± 15.96 (7.95 to 23.34)	0.000*	10.17 ± 9.85 (5.42 to 14.92)	0.000*
		1.0		0.9		0.9		1.0
MIP (% predicted)	20.34 ± 20.35 (9.87 to 30.80)	0.001*	14.04 ± 14.06 (6.55 to 21.53	0.001*	17.42 ± 17.82 (8.83 to 26.01)	0.000*	11.20 ± 10.75 (4.54 to 6.01)	0.000*
		1.0		0.9		0.9		1.0
MEP (cmH_2_O)	25.96 ± 20.23 (15.89 to 36.02)	0.000*	0.87 ± 21.66 (− 10.66 to 12.41)	0.874*	14.75 ± 21.58 (4.35 to 25.15)	0.008**	8.08 ± 22.57 (−2.79 to 18.97)	0.136*
		1.2		0.1				0.3
MEP (% predicted)	23.00 ± 18.23 (13.63 to 32.38)	0.000*	0.95 ± 19.57 (−9.47 to 11.38)	0.848*	13.18 ± 19.00 (−4.02 to 22.34)	0.007**	6.96 ± 20.07 (−2.70 to 16.64)	0.148*
		1.2		0.1				0.3
	Groups N-C and I-C		Groups N-C and N-H		Groups N-H and I-H		Groups I-C and I-H	
MIP (cmH_2_O)	5.77 ± 5.53 (−5.52 to 17.05)	0.305†	2.78 ± 5.72 (−8.84 to 14.40)	0.630†	5.48 ± 4.30 (−3.31 to 14.27)	0.212†	2.49 ± 3.82 (− 5.29 to 10.27)	0.519†
		0.3		0.1		0.4		0.2
MIP (% predicted)	5.38 ± 9.04 (−13.19 to 23.96)	0.556†	2.92 ± 6.36 (− 10.01 to 15.85)	0.649†	6.22 ± 4.76 (−3.54 to 15.98)	0.203†	3.75 ± 7.43 (− 11.37 to 18.87)	0.617†
		0.3		0.1		0.4		0.2
MEP (cmH_2_O)	25.14 ± 7.40 (10.05 to 40.24)	0.002†	11.27 ± 7.09 (−3.15 to 25.68)	0.121††	6.66 ± 7.17 (−7.87 to 21.20)	0.359††	−7.22 ± 7.52 (−22.52 to 8.09)	0.344†
		1.9						0.3
MEP (% predicted)	22.06 ± 6.58 (8.64 to 35.48)	0.002†	9.82 ± 6.22 (−2.83 to 22.48)	0.124††	6.22 ± 6.34 (−6.65 to 19.08)	0.334††	−6.02 ± 6.73 (−19.72 to 7.68)	0.378†
		1.1						0.3

Continuous variables are reported as mean ± standard deviation (95% confidence interval)

*Paired *t*-test. ** Wilcoxon signed-rank test. †Independent *t*-test. †† Mann–Whitney test

***Abbreviations*:** *MIP* maximal expiratory pressure, *MEP* maximal expiratory pressure, *ES* Effect size

### Exercise adherence

There was no significant difference in the training adherence rate between the home- and center-based training groups. Adherence rates in each group ranged from 91 to 99% (Table 1).

### Diaphragm thickness and PCF

Significant increases in the right diaphragm thickness at end-tidal volume were identified in Groups N-C (*P* = 0.026), N-H (*P* = 0.018), and I-H (*P* = 0.022). Groups N-H (*P* = 0.010) and I-H (*P* = 0.007) showed significant improvements in PCF (Table 3).

**Table 3:** Mean changes in secondary outcomes after 8 weeks of inspiratory muscle training

	Group N-C (n = 17)		Group I-C (n = 16)		Group N-H (n = 19)		Group I-H (n = 19)	
Outcomes	Change from baseline	*P* value	Change from baseline	*P* value	Change from baseline	*P* value	Change from baseline	*P* value
		ES		ES		ES		ES
Right diaphragm thickness at end inspiration (mm)	0.70 ± 1.17 (0.09 to 1.30)	0.026*	0.20 ± 1.27 (−0.47 to 0.88)	0.527	0.50 ± 0.83 (0.09 to 0.90)	0.018*	0.62 ± 1.09 (0.10 to 1.15)	0.022*
		0.5		0.1		0.6		0.5
BMI (kg/m^2^)	−0.99 ± 2.85 (−2.46 to 0.47)	0.169	0.78 ± 1.40 (0.00 to 1.56)	0.048*	0.19 ± 0.85 (− 0.21 to 0.60)	0.340	0.23 ± 1.00 (− 0.24 to 0.71)	0.318
		0.3		0.5		0.2		0.2
FVC (% predicted)	2.05 ± 4.09 (0.04 to 4.16)	0.055	3.18 ± 6.96 (−0.52 to 6.90)	0.087	−1.63 ± 19.24 (−10.90 to 7.64)	0.716	0.57 ± 8.00 (−3.28 to 4.43)	0.756
		0.5		0.4		0.1		0.1
FEV1 (% predicted)	−1.00 ± 4.25 (−3.18 to 1.18)	0.347	2.25 ± 14.59 (−5.52 to 10.02)	0.547	4.10 ± 19.80 (− 5.44 to 13.65)	0.378	3.31 ± 10.23 (− 1.61 to 8.24)	0.175
		0.2		0.1		0.2		0.3
PCF (L/min)	6.11 ± 55.00 (−21.24 to 31.46)	0.643	3.12 ± 34.19 (−15.09 to 21.34)	0.720	23.68 ± 35.77 (6.43 to 40.92)	0.010*	29.47 ± 42.48 (8.99 to 49.95)	0.007*
		0.1		0.1		0.6		0.6
RMS (uV)	9.76 ± 78.75 (−5.02 to 24.54)	0.181	6.23 ± 22.36 (− 5.68 to 18.15)	0.283	8.23 ± 21.45 (−2.10 to 18.57)	0.112	7.89 ± 29.96 (−6.54 to 22.34)	0.266
		0.1		0.2		0.3		0.2
SMI	−0.06 ± 0.33 (−0.23 to 0.10)	0.711	0.03 ± 0.34 (− 0.15 to 0.22)	0.711	0.10 ± 0.26 (− 0.02 to 0.22)	0.114	0.03 ± 0.31 (− 0.11 to 0.18)	0.663
		0.1		0.1		0.3		0.1
Bioimpedance-derived phase angle	0.06 ± 0.32 (−0.10 to 0.23)	0.428	0.12 ± 0.32 (− 0.05 to 0.30)	0.152	− 0.08 ± 0.30 (− 0.22 to 0.06)	0.240	0.06 ± 0.30 (−3.28 to 4.43)	0.756
		0.1		0.3		0.2		0.2
IPAQ (MET-min/week)	− 236.82 ± 2294.48 (− 1416.53 to 942.88)	0.676	−229.06 ± 2018.41 (− 1304.60 to 846.47)	0.656	− 165.73 ± 1745.60 (− 1007.09 to 675.61)	0.684	− 277.84 ± 2939.75 (− 1694.75 to 1139.07)	0.685
		0.1		0.1		0.1		0.1
VO_2_ peak (ml/kg/min)	1.44 ± 5.88 (−1.48 to 4.36)	0.312	0.73 ± 5.26 (−2.06 to 3.54)	0.583	−1.64 ± 7.63 (−5.32 to 2.03)	0.361	−1.76 ± 7.38 (− 5.32 to 1.79)	0.312
		0.2		0.1		0.2		0.5

Continuous variables are reported as mean ± standard deviation (95% confidence interval)

***Abbreviations*:** *BMI* body mass index, *FVC* forced vital capacity, *FEV*_*1*_ forced expiratory volume in the first second, *PCF* peak cough flow, *RMS* root mean square, *SMI* skeletal muscle index, *IPAQ* International Physical Activity Questionnaire; VO_2_ peak, peak oxygen uptake

### Adverse events

No adverse events related to the intervention were reported among the participants during the clinical trial, except for one patient with a transient headache. Although this possible mild adverse event appeared at the end of the first training session, the hemodynamic response was normal, and the symptom was relieved within a 5-min break. In this participant, the intensity of the RMT was initiated at 50% of the target and was then gradually increased, with no subsequent symptoms.

## Discussion

### MIP, MEP, and exercise adherence

Until now, no previous randomized, non-inferiority trial has attempted to validate the effect of the newly developed IMT/PEP device for older men. In previous studies, it was typically proposed that RMT should be performed for about 30 min, or two 15-min sessions daily [32–34]. In this study, the session consisted of performing 10 sets of 10 breaths, in consideration of the participant’s compliance and for the ease of calculating the number of repetitions. The protocol involved training for 8 weeks, for two sessions a day. The primary finding of this study was that, after 8 weeks of training, inspiratory muscle strengthening via the new IMT/PEP device was not inferior to that achieved using the Threshold IMT device. Furthermore, the IMT/PEP device resulted in significant improvement in MEP, which is related to the power of expiratory muscles, which was not observed in the Threshold IMT training groups. Of course, significant MEP improvements could have been expected if the additional conventional Threshold PEP device (Philips Respironics Inc., Murrysville, PA, USA) was also included for use by Groups I-C and I-H. However, the use of two separate devices, namely, the Threshold IMT and Threshold PEP, is likely to double the training time and complicate the training procedure. Although multiple factors affect training adherence [35], a previous study has shown that a shorter training time has a positive effect on training adherence [36]. From this perspective, we used only the Threshold IMT device in Groups I-C and I-H to standardize the time spent on training. We hypothesized that simultaneous inspiration and expiration training within a single breath would yield additional benefits. However, there were no statistically significant differences in the mean MIP between groups before and after training, as compared to the results when the conventional Threshold IMT device was used. Furthermore, there were no significant differences in the primary outcome between protocols, which indicated that home-based training is non-inferior to a center-based protocol.

The role of home-based respiratory rehabilitation has been highlighted in the era of the COVID-19 pandemic [37, 38]. Exercise adherence––previously one of the barriers to home-based training––was greater than 90% in all groups in this study. Of course, the high adherence rate seen in those who completed the training may have been a result of some selection bias, but the adherence rates were comparable between protocols. In addition, patient withdrawal from home-based training groups was less than that of center-based training. This result suggested that the lack of information and motivation, one of the barriers to pulmonary rehabilitation, can be overcome by using a single supervised home-based RMT.

### Exercise performance

Several studies have reported an improved effect of RMT on exercise performance in healthy individuals [39–41]. The protocol of alternating inspiratory and expiratory muscle training showed a better effect on exercise performance than a single type of RMT alone [11]. RMT also had a greater effect in less-fit participants, such as older individuals [27, 28]. Although we expected an improvement in exercise performance because of the population targeted, the mean value of the predicted VO_2_ peak showed no significant improvement. We considered that there were two reasons for this result. First, the RMT device used in this study had a low maximum threshold setting and thus could not be sufficiently raised with exercise intensity. Second, various tests, such as the incremental test, constant load test, and time trial, have been used in an attempt to assess the effect of RMT on exercise performance improvement, but significant improvement was confirmed only with the constant load and time trial test [11]. In this study, it was difficult to verify the effect of exercise tolerance because the incremental ramp protocol (the Chester step test) was selected to measure aerobic capacity due to the limitation of the research equipment.

### Diaphragm thickness and muscle mass

Low muscle mass and age-related sarcopenia also affect respiratory muscle strength in older individuals, triggering vulnerability to disease and disability [6]. RMT strengthens and improves the thickness and movement of the diaphragm, which is the main inspiratory muscle [4]. In this study, significant improvements in diaphragm thickness were found in groups N-C, N-H, and I-H.

In BIA, the phase angle is presented as an alternative predictor of health in the aging process [42, 43]. In this study, the initial phase angle exceeded the average value of 5.32 ± 0.62 in the community-dwelling older people that was previously reported [44]. In addition, the skeletal muscle index was higher than the 7.0 kg/m^2^ presented by the Asian Working Group for Sarcopenia [3]. We observed no significant improvements in muscle mass or phase angle in any group. We assume that the initial values were markedly superior, such that the low intensity of RMT did not influence these results. In future, it will be necessary to evaluate these effects on frail older individuals or patients with respiratory diseases.

### Respiratory muscle activation during RMT

Loaded breathing leads to greater activation of the neck muscles [45]. Generally, it is necessary to use accessory respiratory muscles and the diaphragm for effective RMT. Therefore, single-channel sEMG on the left SCM was used in the first and final training sessions to provide visual feedback of accessory muscle activity, as well as an outcome measure. We expected that if the diaphragm was strengthened, activation of the accessory muscles to overcome the same threshold loading would be relatively reduced. However, no significant differences between before and after training were identified in any of the groups. A study of chronic obstructive pulmonary disease (COPD) patients suggested that PEP training reduces the activity of the SCM muscles during respiration, which implies improved respiratory efficiency [46]. In the community-dwelling older individuals with normal lung function, it was difficult to confirm a significant difference in RMS because there was little use of accessory muscles during quiet breathing, and the function of the diaphragm was sufficient to overcome the threshold. However, it can be expected that the combined IMT/PEP training will show a difference in SCM muscle activity in COPD patients.

### Limitations

There were some limitations to this study. First, significant effects, such as increased activity or aerobic capacity, could not be identified due to the low maximum thresholds in the devices used. In our next study, we plan to verify the clinical impact of the IMT/PEP device on patients with chronic lung disease. In addition, due to the limitations of the protocol, there was no re-evaluation within the 8-week intervention. Therefore, we did not readjust the threshold during the training period. Consequently, we expect that the load gradually decreased below 40% of the MIP during training as the respiratory effort became stronger.

## Conclusion

We observed that the IMT/PEP device was non-inferior to and yielded additional effects compared to the verified Threshold IMT. The reduced training time and improved usability, which is associated with exercise adherence, provided further advantages. Furthermore, our results indicated that, if preceded by proper education, a home-based RMT alone with the new IMT/PEP could provide a sufficient effect in older individuals. In addition, the results of this study suggest that the new RMT device can be used as an effective treatment strategy, even in patients with chronic lung diseases that require pulmonary rehabilitation.

## Data Availability

The datasets used and/or analyzed during the current study are available from the corresponding author on reasonable request.

## Abbreviations

BMI: Body mass index
COPD: Chronic obstructive pulmonary disease
FEV_1_: Forced expiratory volume in the first second
FVC: Forced vital capacity
IMT: Inspiratory muscle training
IPAQ: International Physical Activity Questionnaire
MCID: Minimal clinically important significance
MEP: Maximal expiratory pressure
MIP: Maximum inspiratory pressure
PCF: Peak cough flow
PEP: Positive expiratory pressure
RMS: Root mean square
RMT: Respiratory muscle training
sEMG: Surface electromyography
SMI: Skeletal muscle index
VO_2_ peak: Peak oxygen uptake
